# ﻿A new provannid snail (Gastropoda, Abyssochrysoidea) discovered from Northwest Eifuku Volcano, Mariana Arc

**DOI:** 10.3897/zookeys.1112.85950

**Published:** 2022-07-14

**Authors:** Chong Chen, Hiromi Kayama Watanabe

**Affiliations:** 1 X-STAR, Japan Agency for Marine-Earth Science and Technology (JAMSTEC), 2-15 Natsushima-cho, Yokosuka, Kanagawa, 237-0061, Japan X-STAR, Japan Agency for Marine-Earth Science and Technology (JAMSTEC) Yokosuka Japan

**Keywords:** Deep sea, hydrothermal vent, Mollusca, new species, Provannidae, Western Pacific

## Abstract

Gastropods in the family Provannidae are characteristic members of deep-sea chemosynthesis-based communities. Recently, surveys of hydrothermal vents and hydrocarbon seeps in the western Pacific have revealed a high diversity of provannids, with new discoveries continuing to be made. Here, we report and describe a further new species, *Provannaexquisita***sp. nov.**, discovered from the Northwest Eifuku volcano on the Mariana Arc. This new species is distinguished from all other described *Provanna* species by its exaggerated sculpture characterised by two to three sharply raised, flange-like keels on the teleoconch whorls. The status of *P.exquisita***sp. nov.** is also supported by a molecular phylogeny reconstruction using the mitochondrial cytochrome *c* oxidase subunit I (COI) gene, which suggested that it is most closely related to a clade of three species described from Okinawa Trough vents including *P.clathrata*, *P.subglabra*, and *P.fenestrata*. Despite being one of the better-explored regions of the world in terms of hydrothermal vent biodiversity, new discoveries like *P.exquisita***sp. nov.** continue to remind us that we are nowhere near fully documenting the species diversity in these unique ecosystems—despite the species being threatened from imminent anthropogenic impacts such as deep-sea mining.

## ﻿Introduction

Hydrothermal vent ecosystems in the deep sea host lush biological communities sustained by microbial chemosynthesis using hydrogen sulfide and other reducing substances dissolved in the vent fluid. First discovered in 1977 on the Galápagos Rift (Corliss et al. 1979), over 300 active vents have been confirmed around the world, concentrated on mid-ocean ridges, volcanic arcs, and back-arc basins (Beaulieu and Szafranski 2020). The Izu-Ogasawara (Bonin)-Mariana (IBM) Arc in the western Pacific is home to over a dozen known active vent sites, typically located on submarine volcanoes (Watanabe et al. 2019). One of these is the Northwest Eifuku (NW Eifuku) Volcano, hydrothermal activity on which was discovered during the NOAA Ocean Exploration Program’s “Submarine Ring of Fire” (SROF) project that undertook surveys of a number of volcanoes on the Mariana Arc from 13.5°N to 22.5°N (Embley et al. 2007). The hydrothermally active sites at the summit of NW Eifuku discovered in March and April 2004 are notable for the presence of cold liquid carbon dioxide (CO_2_) discharge in addition to hot hydrothermal fluid venting from white smokers at the Champagne vent (Lupton et al. 2006). This CO_2_ flux leads to an extreme habitat where dense animal colonies dominated by the bathymodioline mussel *Bathymodiolusseptemdierum* Hashimoto & Okutani, 1994 are found, with pH as low as 5.36 (Limén and Juniper 2006; Tunnicliffe et al. 2009; Rossi and Tunnicliffe 2017).

Gastropod molluscs are prevailing inhabitants of vent ecosystems (Warén and Bouchet 1993, 2001) and about two-thirds of gastropods found at vents occur in no other environments (Wolff 2005). Provannidae is a gastropod family found exclusively in chemosynthesis-based ecosystems (Chen et al. 2019; Linse et al. 2019), recently revealed to be paraphyletic due to genera in the closely related family Abyssochrysidae becoming nested with genera considered to be provannids in phylogenetic reconstructions (Johnson et al. 2010; Souza et al. 2020). The two families together form the superfamily Abyssochrysoidea (Souza et al. 2020). Currently, four genera from chemosynthetic ecosystems, including the endosymbiotic *Alviniconcha* and *Ifremeria*, as well as the non-symbiotic *Provanna* and *Desbruyeresia*, are assigned to Provannidae, two genera including *Abyssochrysos* from non-chemosynthetic deep sea and *Cordesia* from organic falls are assigned to Abyssochrysidae, while *Rubyspira* from organic falls remains unassigned to either family (Souza et al. 2020). Familial affinities of the genera still remain in a state of flux.

*Provanna* is the most species-rich abyssochrysoid genus, with 27 described species inhabiting hot vents, cold seeps, and organic falls between 450–5687 m deep around the globe (Sasaki et al. 2010; Linse et al. 2019). Recent explorations of vents and seeps in the western Pacific have revealed a high diversity of *Provanna* species (Sasaki et al. 2016; Chen et al. 2019; Ke et al. 2022). Here, we report a further previously undescribed species of *Provanna* with a striking sculpture, discovered from the hydrothermal vent field near the summit of NW Eifuku Volcano, providing a formal description and testing its relationship with other abyssochrysoid species using molecular phylogenetic reconstruction with the mitochondrial cytochrome *c* oxidase subunit I (COI) gene.

## ﻿Materials and methods

### ﻿Sample collection

**Figure 1.**
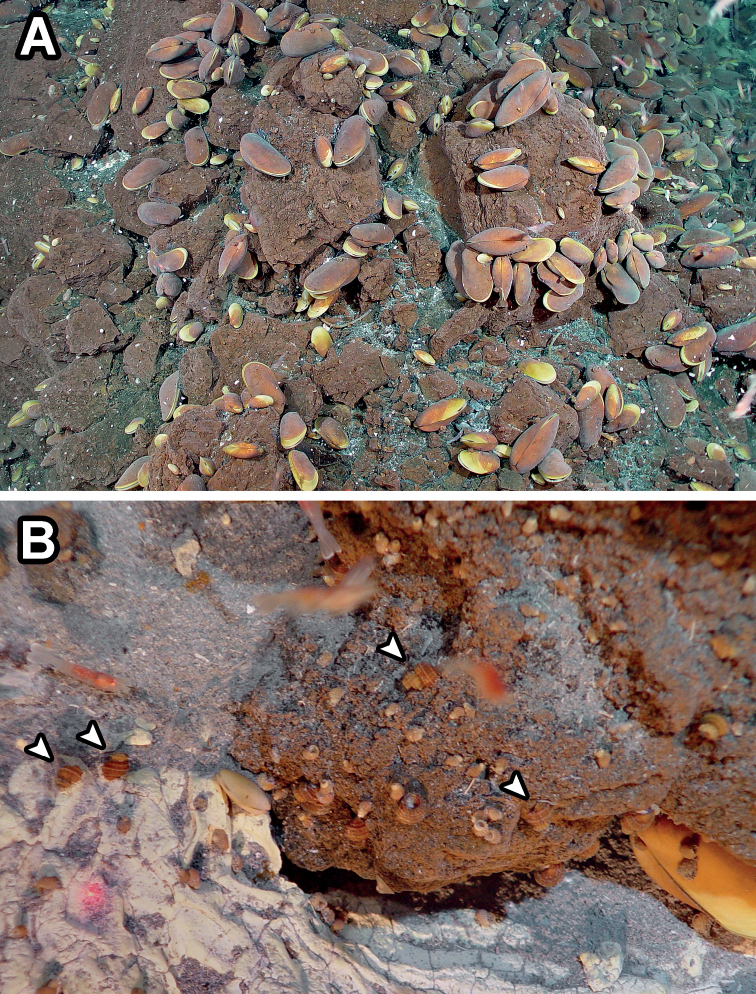
*In situ* habitat of *Provannaexquisita* sp. nov. on the summit of Northwest Eifuku Volcano. **A** overview of the habitat showing dominance by the deep-sea mussel *Bathymodiolusseptemdierum***B** close-up of the mixed provannid aggregation, arrowheads indicate examples of *P.exquisita* sp. nov. Photos taken by ROV*ROPOS* on dive #792 in Champagne vent, 21°29.25'N, 144°02.49'E, 1608 m deep.

Provannid snails were collected from near the summit of NW Eifuku Volcano, Mariana Arc (Fig. 1) using the remotely-operated vehicle (ROV) *JASON II* on-board the R/V “Roger Revelle” cruise RR141 “Submarine Ring of Fire 2014 – Ironman” (chief scientist Craig Moyer and William Chadwick). Upon recovery on deck, snails were sorted from the biological material collected and preserved in 75% ethanol until further investigation in the laboratory. *In situ* images of NW Eifuku were taken using a video camera on ROV*JASON II* and supplemented by high-resolution photographs taken by a digital still camera of ROV*ROPOS* on-board the R/V “Thomas G. Thompson” cruise TN167 “Submarine Ring of Fire 2004” (chief scientist Robert W. Embley).

### ﻿Morphology

Provannid snails were observed and dissected under an Olympus SZX7 dissecting microscope and photographs were taken using a digital single reflex camera (Olympus OM-D E-M5 Mark III) mounted on the trinocular. For specimen photos, several photos taken at different focus levels were stacked automatically using Adobe Photoshop 2022 software. Shell height (SH), shell width (SW), aperture height (AH), and aperture width (AW) were measured using digital Vernier callipers, with the values rounded up to the nearest 0.1 mm. In specimens with a damaged aperture, only SH and SW were taken.

### ﻿Electron microscopy

The radula was dissected from the radula sac using fine tweezers and placed in 5% sodium hypochlorite solution to dissolve any remaining soft tissue, for about 5 min. The operculum was dissected and sulfide deposits on the surface were cleaned off using a soft brush. The radula and operculum were washed twice in Milli-Q water before mounting on aluminium stubs using carbon tape for scanning electron microscopy (SEM). A tabletop SEM (Hitachi TM3000) was used for observation and imaging of the radula and operculum.

### ﻿DNA extraction and sequencing

Genomic DNA was extracted from a section of the provannid snail’s foot musculature using the QIAGEN DNeasy Blood and Tissue Kit (QIAGEN, Tokyo, Japan) following the manufacturer’s standard instructions and then purified using GeneReleaser (BioVentures Inc., Marfreesboro, USA) also following the manufacturer’s protocol. The quality of the extracted DNA was checked using a Thermo Scientific NanoDrop 2000 spectrophotometer. The *Provanna*-specific primer pair for the mitochondrial COI gene, Pg394L (5’-CTGATTTTTCGGACATCCTG-3’) and Pg1253R (5’-TGTTGAGGAAAGAAAGTAATATTAA-3’) were used for amplification via polymerase chain reaction (PCR) in a 20 μl reaction volume consisting of 1 μl template DNA, 1 μl of each primer, 10 μl of Premix *ExTaq* HS DNA polymerase (TaKaRa, Shiga, Japan) and 7 μl de-ionized sterilized water. A Veriti Thermal Cycler (Applied Biosystems) was used for PCR with the following protocol: 94 °C for 2 min followed by 30 cycles of (94 °C for 30 s, 45 °C for 30 s, 72 °C for 30 s), ending with 72 °C for 90 s. The successful PCR product was purified using ExoSAP-IT (Affymetrix) following standard protocols and submitted to FASMAC Corporation (Kanagawa, Japan) for Sanger sequencing. Sequencing was done using the universal primer HCO2198 (Folmer et al. 1994) and the *Provanna*-specific Pg696R (5’-CAGGATGTCCGAAAAATCAG- 3’) in addition to Pg394L and Pg1253R.

### ﻿Molecular analyses

Geneious Prime 2021.2.2 (https://www.geneious.com/) was used to align and manually correct the sequences obtained into a consensus sequence, deposited in GenBank under the accession number ON324570. This newly generated sequence of the NW Eifuku *Provanna* and a sequence of *Cordesiaatlantica* Souza, Passos, Shimabukuro & Sumida, 2020 (Souza et al. 2020) was added to a 530-bp alignment with other abyssochrysoid COI sequences available on GenBank used in a previous publication by Linse et al. (2019), using the MUSCLE alignment in Geneious. Four non-abyssochrysoid caenogastropods were used as outgroups, including two cerithiids, *Cerithiumzonatum* (Wood, 1828) and *Bittiolumvarium* (Pfeiffer, 1840), and two littorinids, *Littorinalittorea* (Linnaeus, 1758) and *Echinolittorinavidua* (Gould, 1859).

Phylogenetic reconstruction was conducted using Bayesian inference with MrBayes v. 3.2 (Ronquist et al. 2012), using the nucleotide substation models HKY+I+G for the first and second codon positions and GTR+I+G for the third codon position selected by the Bayesian information criterion in PartitionFinder v. 2.1.1 (Lanfear et al. 2016). Markov chain Monte Carlo chains were run for 1 million generations with topologies being sampled every 100 generations. The first 25% of trees were discarded as “burn-in” and convergence was checked using the software Tracer v. 1.7 (Rambaut et al. 2018). The Kimura-2-parameter (K2P) distance (Kimura 1980) between COI sequences of different *Provanna* species was calculated using the package MEGA X (Kumar et al. 2018) using the same alignment.

### ﻿Specimen repository

Specimens examined in the present study were deposited in public museum collections, including Museum national d’Histoire naturelle (MNHN), Paris, France and the National Museum of Nature and Science, Tsukuba (NSMT), Japan.

## ﻿Results

### ﻿Systematics

**Subclass Caenogastropoda Cox, 1960**

**Superfamily Abyssochrysoidea Tomlin, 1927**

#### Family Provannidae Warén & Ponder, 1991

##### ﻿ Provanna

Taxon classificationAnimaliaLittorinimorphaProvannidae

Dall, 1918

2AF9493C-F3B4-5A2C-A439-84827F911561

###### Type species.

*Provannalomana* (Dall, 1918).

##### ﻿ Provanna exquisita sp. nov.

Taxon classificationAnimaliaLittorinimorphaProvannidae

DA1C0F34-2688-584B-8BCF-BB334FBCA665

https://zoobank.org/57593DBE-5809-41CC-B5A7-AA1A5726A1F7

Figs 2A–D , 3A–D

Provanna aff. fenestrata —Giguère and Tunnicliffe 2021: supplementary table S2

###### Type locality.

Hydrothermal vent near the summit of Northwest Eifuku Volcano (Lupton et al. 2006; Rossi 2016) on the Mariana Arc; 21°29.2567'N, 144°02.4813'E, 1606 m deep (“Golden Lips” site 40 m away from the Champagne vent). Habitat temperature 2.7 °C, sulfide level negligible. ROV*JASON II* dive #799, 2014/xii/14, R/V *Roger Revelle* cruise RR1413 “Submarine Ring of Fire 2014 – Ironman”.

###### Type material.

**Figure 2.**
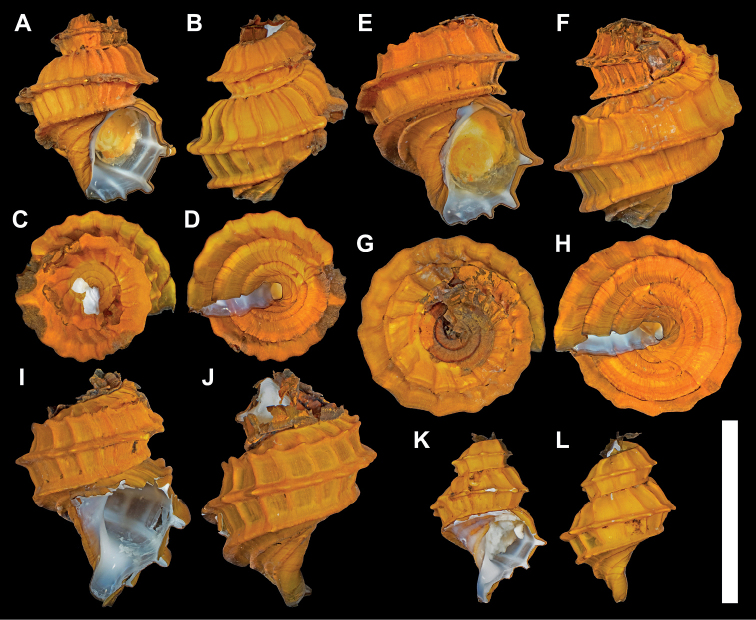
*Provannaexquisita* sp. nov., type specimens **A–D** holotype (MNHN-IM-2000-37945), shell height 10.4 mm **E–H** paratype #1 (NSMT-Mo 79360), shell height 11.6 mm **I, J** paratype #2 (MNHN-IM-2000-37946), shell height 13.1 mm **K, L** paratype #3 (NSMT-Mo 79361), shell height 9.4 mm. Scale bar: 1 cm, applies to all parts of the figure.

**Figure 3.**
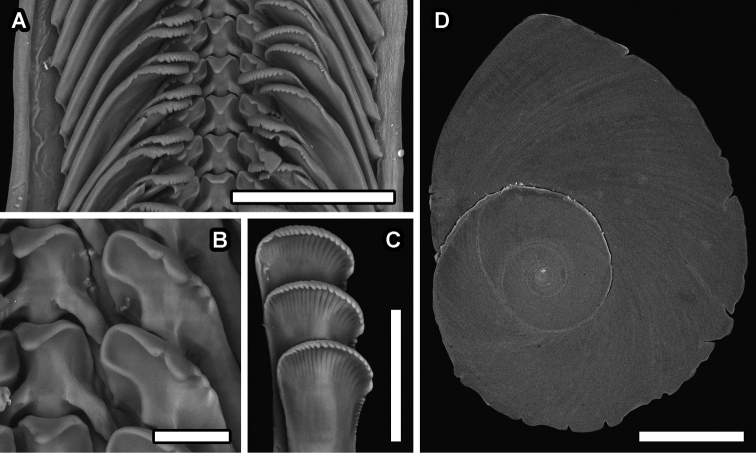
*Provannaexquisita* sp. nov., scanning electron micrographs **A** radula overview **B** close-up of central and lateral teeth **C** close-up of marginal cusps **D** operculum. Scale bars: 100 μm (**A**); 20 μm (**B**); 50 μm (**C**); 1 mm (**D**).

***Holotype*** (Fig. 2A), MNHN-IM-2000-37945; SH 10.4 mm, SW 9.0 mm, AH 6.1 mm, AW 4.5 mm. ***Paratype*** #1 (Fig. 2B), NSMT-Mo 79360; SH 11.6 mm, SW 10.3 mm, AH 7.1 mm, AW 5.6 mm. ***Paratype*** #2 (Fig. 2C, Fig. 3A-D), MNHN-IM-2000-37946; SH 13.1 mm, SW 9.4 mm; aperture damaged, soft parts extracted and used for DNA barcoding and dissected for SEM. ***Paratype*** #3 (Fig. 2D), NSMT-Mo 79361; SH 9.4 mm, SW 7.2 mm; aperture broken. All type material were live collected from the type locality and preserved in 75% ethanol.

###### Diagnosis.

A large *Provanna* reaching over 13 mm in shell height (exceeds 15 mm if spire intact), teleoconch whorls with two or three sharply raised, flange-like spiral keels crossing with weaker axial ribs to form a regularly latticed sculpture.

###### Description.

Shell (Fig. 2). Teleoconch thin and fragile, translucent, thickened where ribs or keels occur. Whorls highly convex, inflated for its genus. Suture distinct, well defined, impressed. Spiral sculpture of 2 or 3 very strong, sharply raised, approximately equally-spaced blade or flange-like keels, positioned at shoulder, mid-whorl, just above suture. Some individuals lack shoulder keel; other 2 always present. Three additional weaker spiral ribs present anterior to suture. Axial sculpture of 14–18 regularly spaced, raised ribs running from suture to suture, approximately equal in strength. Together two directions of ribs intersect to form regular reticulate sculpture of regular rectangles. Nodes drawn out on spiral keels where intersection with axial ribs occur, resulting in undulated edges, on shoulder keel these develop into short spines. Aperture semicircular, taller than wide. Columellar variable from straight to sigmoidal. Siphonal notch distinct, shallow. Apex decollate, heavily corroded, leaving only 1.5–2.5 whorls of teleoconch whorls. Incompletely corroded periostracum present around apex, darkened in colouration. Secondary plug-like calcareous secretion present at apex, preventing exposure of visceral mass. Growth lines indistinct. Protoconch unknown, as all specimens examined had corroded spire.

Periostracum thick, golden brown.

Operculum (Fig. 3D) present. Paucispiral, oval, bluntly pointed. Nucleus eccentric, 3.5 whorls. Yellowish-brown in colour, thin, semitransparent.

Radula (Fig. 3A–C) taenioglossate, formula 2 + 1 + 1 + 1 + 2. Central tooth solid, with single triangular, bluntly pointed, overhanging main cusp. Solid lateral support ridges present on both sides of central supporting ridge. Shaft with 2 sharp protrusions at anterior edge of lateral support ridge. Lateral teeth solid, inner edge sigmoidal. Laterals with 5 cusps, main cusp triangular, bluntly pointed. One moderately strong inner cusp inside main cusp, 3 weaker cusps outside main cusp. Sharply raised protrusion present on shafts of laterals below main cusp. Marginal teeth flat, broad, truncated distally. Distal tip rake-like, finely serrated into c. 22–24 denticles, outermost strongest. Further c. 10–12 minor denticles present on outermost cutting edge, below strongest cusp.

Gross external anatomy examined to limited extent with 2 brittle, ethanol-preserved specimens, revealing no notable deviations from published accounts for its genus (Warén and Ponder 1991; Chen et al. 2019). Animal occupied approximately 1.5–2 whorls. Head with flattened snout, 1 pair of equally sized, tapering cephalic tentacles present; eyes lacking. Penis, neck furrow, epipodial tentacles lacking. Pallial edge smooth. Gill monopectinate, typical for its genus, not hypertrophied. Apex of visceral mass occupied by gonad, posterior of digestive gland.

###### Distribution.

So far, it is only known from a hydrothermal vent field on the summit of Northwest Eifuku Volcano, Mariana Arc. In addition to the Golden Lips site where specimens were collected, it has also been visually confirmed from the Champagne site 40 m away (Fig. 1B).

###### Etymology.

*Exquisita* (Latin, feminine adjective in the nominative singular), meaning “inquiring” or “exquisite”.

###### Remarks.

The striking shell sculpture of *Provannaexquisita* sp. nov., especially the prominent sharply raised spiral keels, is unique among described *Provanna* species. The species with the closest morphology is *Provannafenestrata* Chen, Watanabe & Sasaki, 2019 described from Okinawa Trough vents and also recently reported from a methane seep in the South China Sea (Chen et al. 2019; Ke et al. 2022), which also has a similar coarse, regular, lattice-like sculpture. In *P.fenestrata*, however, the spiral and axial ribs are of similar strength and spiral ribs do not form raised keels; nodes at the intersection between the two nodes are also lacking in *P.fenestrata* (Chen et al. 2019). In individuals of *P.fenestrata* with 2 spiral ribs, it is always the mid-whorl spiral rib that is missing, whereas the missing spiral keel is always the posterior-most shoulder keel in *P.exquisita* sp. nov. Furthermore, the periostracum of *P.fenestrata* is yellowish green compared to golden brown in *P.exquisita* sp. nov. The radulae of the two species are similar, although in *P.fenestrata* the central and lateral teeth have sharper cusps and the marginals are less serrated (12–14 vs 22–24 denticles).

A number of other *Provanna* species also exhibit reticulate shell sculpture, including *P.clathrata* Sasaki, Ogura, Watanabe & Fujikura, 2016 from Okinawa Trough vents, *Provannapacifica* (Dall, 1908) from seeps in Gulf of Panama and Oregon Margin, *Provannamuricata* Warén & Bouchet, 1986 from Galápagos Rift vents, *P.admetoides* Warén & Ponder, 1991 from Florida Escarpment seeps, *P.segonzaci* Warén & Ponder, 1991 from Lau Basin vents, *Provannabuccinoides* Warén & Bouchet, 1993 from Lau and North Fiji vents, and *Provannareticulata* Warén & Bouchet, 2009 from seeps off West Africa. However, compared to *P.exquisita* sp. nov. all of these species exhibit much weaker spiral sculpture (Warén and Bouchet 1986, 2001, 2009; Warén and Ponder 1991; Sasaki et al. 2016). The spiral ribs of *P.muricata* are much weaker than the axial ones, which is opposite to the pattern seen in *P.exquisita* sp. nov. (Warén and Bouchet 1986). The radulae of *P.pacifica* and *P.admetoides* exhibit slender, reduced central teeth and are very different from the solid central tooth in *P.exquisita* sp. nov. (Warén and Bouchet 1986; Warén and Ponder 1991).

### ﻿Molecular support

**Figure 4.**
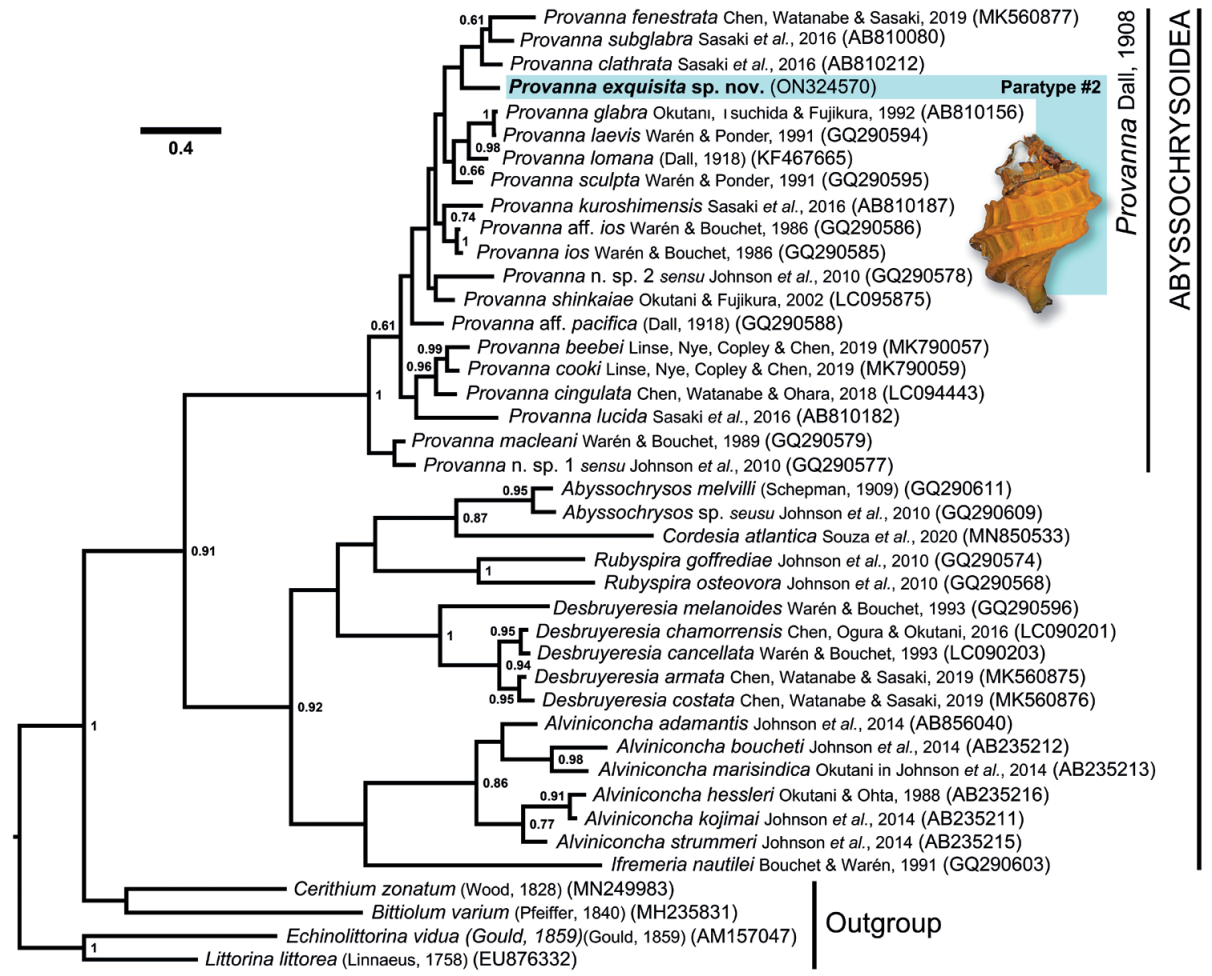
Bayesian phylogenetic reconstruction of Abyssochrysoidea using a 530-bp alignment of partial COI sequences. Node values are Bayesian posterior probabilities, those less than 0.6 not shown. Scale bar indicates substitutions per site.

The phylogenetic tree of the superfamily Abyssochrysoidea reconstructed with Bayesian inference using the mitochondrial COI gene is shown in Fig. 4. The phylogeny recovered all seven currently recognised genera in Abyssochrysoidea (*Provanna*, *Desbruyeresia*, *Abyssochrysos*, *Cordesia*, *Alviniconcha*, *Ifremeria*, *Rubyspira*) as monophyletic clades with weak to full support (Bayesian posterior probability (BPP) between 0.46–1). *Provanna* which was fully supported (BPP = 1) as the earliest-branching genus within the superfamily. Within *Provanna*, *P.exquisita* sp. nov. was recovered sister to a weakly supported clade (BPP = 0.48) containing three species known from Okinawa Trough and South China Sea, including *P.fenestrata*, *P.clathrata*, and *P.subglabra* Sasaki, Ogura, Watanabe & Fujikura, 2016 (Xu et al. 2016; Ogura et al. 2018; Chen et al. 2019; Ke et al. 2022). The clade containing these four species, including *P.exquisita* sp. nov., was also only weakly supported (BPP = 0.58). The K2P genetic distances of *P.exquisita* sp. nov. from *P.clathrata*, *P.subglabra*, and *P.fenestrata* over a 357 bp alignment of the COI gene were 6.65%, 6.60%, and 7.40%, respectively.

## ﻿Discussion

The phylogenetic reconstruction herein recovered Provannidae as a paraphyletic clade, agreeing with previous studies (Johnson et al. 2010; Linse et al. 2019; Souza et al. 2020). Abyssochrysidae, as defined in Souza et al. (2020) to contain *Abyssochrysos* and *Cordesia*, was recovered as monophyletic. The position of genera in our COI tree generally agreed with the 2-gene tree of Souza et al. (2020), with the exception that the symbiotic genera *Alviniconcha* and *Ifremeria* came out as sisters in our tree. Both these trees suggested *Provanna* as the earliest-branching genus in Abyssochrysoidea, but this differs from the 10-gene (3 mitochondrial and 7 nuclear) tree by Breusing et al. (2020), which suggests that the earliest split in the superfamily is between the symbiotic *Alviniconcha–Ifremeria* clade and all the rest. This sister-relationship between the two symbiotic genera found by Breusing et al. (2020) was also recovered in our tree. Though the nodes of the Breusing et al. (2020) tree were much better supported, *Cordesia* was not included in the analyses. The true phylogenetic relationships among abyssochrysoid genera, therefore, require further studies using at least multiple genes from all seven genera, as well as more outgroup taxa, to include potentially closely related lineages from Cerithoidea and Littorinimorpha. Detailed anatomy at the species and genus level may shed further lights on their evolutionary relationships.

*Provannaexquisita* sp. nov. is a large species for its genus. If the spires of specimens examined were intact it would be similar in size or even exceed that of the largest known *Provanna* species, *P.cingulata* Chen, Watanabe & Ohara, 2018 from a serpentinite-hosted system in Mariana Trench, which is known to reach 16.5 mm with a slightly corroded spire (Chen et al. 2018). Although the Golden Lip site where the examined specimens were recovered from is 40 m away from the centre of venting activity and CO_2_ flux at the Champagne vent (Rossi 2016), it still shares with Champagne a similar acidic environment with pH of 5.78 (Rossi 2016; Rossi and Tunnicliffe 2017). This explains the very corroded spire of *P.exquisita* sp. nov., similar to shell dissolution seen in the co-occurring mussel *Bathymodiolusseptemdierum* (Tunnicliffe et al. 2009) and shows that *Provanna* is evidently capable of living in such acidic conditions. Nevertheless, increased energetic demands associated with high pCO_2_ may have impacted its capacity in shell repair and maintenance.

The discovery of *Provannaexquisita* sp. nov. from the Mariana Arc adds to the diversity of known abyssochrysoid from hydrothermal vents on the IBM Arc. In contrast, on the Izu-Ogasawara (Bonin) Arcs, only two provannid genera, *Desbruyeresia* and *Alviniconcha*, have been reported despite considerable sampling efforts (Fujiwara et al. 2013; Chen et al. 2019; Watanabe et al. 2019; Giguère and Tunnicliffe 2021). The three segments of the IBM Arc are separated by the Sofugan Tectonic Line (29°30'N) between Izu and Ogasawara arcs and the conjunction with the West Mariana Ridge (23°N) between Ogasawara and Mariana arcs (Stern et al. 2003). Previous research has shown that the Sofugan Tectonic Line acts as a boundary for faunal subdivision (Watanabe et al. 2019), and it is possible that the conjunction with the West Mariana Ridge also acts similarly, preventing the dispersal of some taxa like *Provanna* with lecithotrophic development (Warén and Bouchet 1993; Chen et al. 2018). Nevertheless, even on the Mariana Arc, *Provanna* is so far only recorded from NW Eifuku and nowhere else (Giguère and Tunnicliffe 2021), and it is possible that further exploration of the IBM Arc vents will reveal other populations and species. Despite being one of the better explored regions of the world in terms of hydrothermal vent biodiversity, new discoveries like *P.exquisita* sp. nov. continue to remind us that some of the species diversity remains undocumented at western Pacific vents—despite many endemic species there being threatened from anthropogenic impacts such as deep-sea mining (Thomas et al. 2022).

## Supplementary Material

XML Treatment for Provanna

XML Treatment for Provanna exquisita

## Supplementary materials

Supplementary material 1 KML file of the type locality of Provannaexquisita sp. nov. Data type Occurence Explanation note KML file of Golden Lip site, NW Eifuku Volcano, the type locality of *Provannaexquisita* sp. nov. https://binary.pensoft.net/file/714992This dataset is made available under the Open Database License (http://opendatacommons.org/licenses/odbl/1.0/). The Open Database License (ODbL) is a license agreement intended to allow users to freely share, modify, and use this Dataset while maintaining this same freedom for others, provided that the original source and author(s) are credited.Chong Chen, Hiromi Kayama Watanabe
